# Toward Harmonizing Quantification of Dopamine Neuron Imaging Biomarkers in Parkinson's Disease: The Centamine Scale

**DOI:** 10.1002/ana.78116

**Published:** 2026-01-20

**Authors:** Zhen Fan, Graham Searle, Gaia Rizzo, Justin Albani, Patrick Cella, Robert Comley, Gregory Klein, Luca Passamonti, Cristian Salinas, Adam J. Schwarz, Leonardo Iaccarino, Gilles Tamagnan, Jamie Eberling, Ken Marek, John Seibyl, Roger N. Gunn, The Parkinson's Progression Markers Initiative, Kenneth Marek, Caroline Tanner, Tanya Simuni, Andrew Siderowf, Douglas Galasko, Lana Chahine, Christopher Coffey, Kalpana Merchant, Kathleen Poston, Roseanne Dobkin, Tatiana Foroud, Brit Mollenhauer, Dan Weintraub, Ethan Brown, Karl Kieburtz, Mark Frasier, Todd Sherer, Sohini Chowdhury, Roy Alcalay, Aleksandar Videnovic, Duygu Tosun‐Turgut, Werner Poewe, Susan Bressman, Jan Hammer, Raymond James, Ekemini Riley, John Seibyl, Leslie Shaw, David Standaert, Sneha Mantri, Nabila Dahodwala, Michael Schwarzschild, Connie Marras, Hubert Fernandez, Ira Shoulson, Helen Rowbotham, Paola Casalin, Claudia Trenkwalder, Todd Sherer, Sohini Chowdhury, Mark Frasier, Jamie Eberling, Katie Kopil, Alyssa O'Grady, Maggie McGuire Kuhl, Leslie Kirsch, Tawny Willson, Charles Adler, Roy Alcalay, Amy Amara, Paolo Barone, Bastiaan Bloem, Susan Bressman, Kathrin Brockmann, Norbert Brüggemann, Lana Chahine, Kelvin Chou, Nabila Dahodwala, Alberto Espay, Stewart Factor, Hubert Fernandez, Michelle Fullard, Douglas Galasko, Robert Hauser, Penelope Hogarth, Shu‐Ching Hu, Michele Hu, Stuart Isaacson, Christine Klein, Rejko Krueger, Mark Lew, Zoltan Mari, Connie Marras, Maria Jose Martí, Nikolaus McFarland, Tiago Mestre, Brit Mollenhauer, Emile Moukheiber, Alastair Noyce, Njideka Okubadejo, Sarah O'Shea, Rajesh Pahwa, Nicola Pavese, Werner Poewe, Ron Postuma, Giulietta Riboldi, Lauren Ruffrage, Javier Ruiz Martinez, David Russell, Marie H Saint‐Hilaire, Neil Santos, Wesley Schlett47, Ruth Schneider, Holly Shill, David Shprecher, Tanya Simuni, David Standaert, Leonidas Stefanis, Yen Tai, Caroline Tanner, Arjun Tarakad, Eduardo Tolosa PhD34, Aleksandar Videnovic, Susan Ainscough, Courtney Blair, Erica Botting, Isabella Chung, Kelly Clark, Ioana Croitoru, Kelly DeLano, Iris Egner, Fahrial Esha, May Eshel, Frank Ferrari, Victoria Kate Foster, Alicia Garrido, Madita Grümmer, Bethzaida Herrera, Ella Hilt, Chloe Huntzinger, Raymond James, Farah Kausar, Christos Koros, Yara Krasowski60, Dustin Le, Ying Liu, Taina M. Marques, Helen Mejia Santana, Sherri Mosovsky, Jennifer Mule, Philip Ng, Lauren O'Brien, Abiola Ogunleye, Oluwadamilola Ojo, Obi Onyinanya, Lisbeth Pennente, Romina Perrotti, Michael Pileggi, Ashwini Ramachandran, Deborah Raymond, Jamil Razzaque, Shawna Reddie, Kori Ribb, Kyle Rizer, Janelle Rodriguez, Stephanie Roman, Clarissa Sanchez, Cristina Simonet, Anisha Singh, Elisabeth Sittig, Barbara Sommerfeld, Angela Stovall, Bobbie Stubbeman, Alejandra Valenzuela, Catherine Wandell, Diana Willeke, Karen Williams, Dilinuer Wubuli

**Affiliations:** ^1^ Xing Imaging‐A Mitro Company London UK; ^2^ GE HealthCare Arlington Heights Illinois USA; ^3^ AbbVie Chicago Illinois USA; ^4^ F. Hoffmann‐La Roche Basel Switzerland; ^5^ Lantheus Medical Imaging Billerica Massachusetts USA; ^6^ Takeda Pharmaceuticals International Cambridge Massachusetts USA; ^7^ Eli Lilly and Company Indianapolis Indiana USA; ^8^ Xing Imaging‐A Mitro Company New Haven Connecticut USA; ^9^ The Michael J. Fox Foundation Research Resources New York New York USA; ^10^ Institute for Neurodegenerative Disorders New Haven Connecticut USA; ^11^ Department of Brain Sciences, Faculty of Medicine Imperial College London London UK

## Abstract

**Objective:**

Dopaminergic imaging is a key biomarker for both the investigation of the biology of Parkinson's disease and related synucleinopathies and the evaluation of potential therapies in clinical trials. This work presents a harmonized approach for quantifying dopaminergic molecular imaging tracers, such as [^123^I]ioflupane (dopamine transporter scan [DaTscan]) single photon emission computed tomography (SPECT) and [^18^F]AV133 positron emission tomography (PET), which assess dopaminergic neuronal loss. The proposed method aims to standardize regional outcome measures using a unified scale called Centamines.

**Methods:**

The Centamines framework comprises 3 analysis levels. Level 1 defines the Centamine scale based on healthy subject data from [^123^I]ioflupane SPECT (n = 224). Level 2 uses head‐to‐head data between Tracer X and [^123^I]ioflupane SPECT to map Tracer X onto the Centamine scale. Level 3 maps additional tracers using prior mappings. A level 2 analysis was performed using [^123^I]ioflupane SPECT and [^18^F]AV133 PET data (n = 68) to convert [^18^F]AV133 PET into Centamines.

**Results:**

Level 1 successfully established the Centamine scale using healthy [^123^I]ioflupane SPECT scans. Level 2 revealed moderate‐strong linear correlations (*R* ^2^ = 0.51–0.83) between [^123^I]ioflupane SPECT and [^18^F]AV133 PET across 5 brain regions. Mapped Centamine values showed minimal differences between tracers, ranging from 1.5% (post‐commissural putamen) to 3% (caudate).

**Interpretation:**

The Centamine scale holds promise for the harmonized quantification of dopaminergic neuronal imaging markers. The Centamine strategy would enable and accelerate clinical trials in Parkinson's disease using dopaminergic imaging outcomes. ANN NEUROL 2026;99:949–963

Recently, data from the Parkinson Progression Marker initiative (PPMI) have demonstrated that the α‐synuclein seed amplification assay coupled with dopamine transporter (DAT) imaging provide a biomarker driven definition for Parkinson's disease (PD).^1^ This has led to the introduction of the concept of neuronal synuclein disease (NSD) encompassing PD, dementia with Lewy Bodies and rapid eye movement (REM) sleep behavior disorder (RBD) and the NSD‐Integrated Staging System (NSD‐ISS^2^). A similar proposal by the SynNeurGe group also supports a biologic classification for PD.^3^ A key goal for the biologic definition and staging is to establish biomarker strategies that inform and accelerate therapeutic trials at all stages of disease.

Dopaminergic imaging biomarkers are well established for PD. Among them, the DAT imaging agent [^123^I]ioflupane single‐photon emission computed tomography (SPECT) is the most widely used,^4^, ^5^, ^6^ with DAT positron emission tomography (PET) imaging agents such as [^18^F]FPCIT^7^, ^8^ and [^18^F]PE2I^9^, ^10^ also routinely deployed in research studies. More recently, [^18^F]AV133, a PET biomarker targeting vesicular monoamine transporter 2 (VMAT2^11^, ^12^, ^13^), has shown enhanced sensitivity for longitudinal monitoring of dopaminergic integrity.^14^

In a related field, recent efforts focused on amyloid and tau imaging has developed a harmonized molecular imaging quantification approach. In 2015, the Centiloid quantification strategy was introduced^15^ for the harmonized analysis of amyloid PET tracers including [^11^C]PIB, [^18^F]florbetapir, [^18^F]flutemetamol, and [^18^F]florbetaben. This was the first time a universal quantification method had been developed for different molecular imaging agents targeting the same biological entity. It has been highly successful in improving the deployment and utility of amyloid imaging in Alzheimer's disease (AD) clinical trials.^16^ By enabling use of multiple tracers, the method has facilitated the execution of large‐scale trials while providing a standardized and easily interpretable scale for eligibility assessment and longitudinal data reporting.^17^, ^18^

Building on the success of Centiloids, the Critical Path for Alzheimer's Disease (CPAD) has spearheaded the development of a similar approach for tau quantification, known as Centaurs.^19^ This framework harmonizes quantification across established tau PET tracers, further enabling their use in clinical trials and enhancing their future clinical utility.

Given these advances, establishing a harmonized quantification approach for dopamine imaging agents would significantly enhance their application in PD clinical therapeutic trials, potentially improving the accuracy of cohort recruitment and assessment of treatment efficacy. This paper introduces the Centamine scale, detailing its framework and methodology, and presents data from [^123^I]ioflupane SPECT and [^18^F]AV133 PET to illustrate the approach. In addition, access to the necessary data is provided, enabling individual labs to calibrate their image analysis pipelines and generate outputs in Centamines.

## Methods

### Data Sets

The data sets used in this work are part of the PPMI study (http://ppmi-info.org), which was conducted in accordance with the Declaration of Helsinki and the Good Clinical Practice (GCP) guidelines after approval of the local ethics committees of the participating sites. All [^123^I]ioflupane SPECT and [^18^F]AV133 PET data were acquired according to the PPMI protocols ([^123^I]ioflupane SPECT: dose: 111–185 MBq, acquisition: 30 minutes at 4 hours post injection; [^18^F]AV133 PET: dose: 185 MBq +/− 10%, acquisition: 10 minutes at 80 minutes post injection.^20^, ^21^ This analysis used data openly available from PPMI (tier 1).

- Data set 1 (healthy [^123^I]ioflupane SPECT): 227 healthy individuals (sex, 137 males/90 females; age, 30–85 years; mean [m], 62.0; standard deviation [sd], 11.8 years) were imaged with [^123^I]ioflupane SPECT.
- Data set 2 (head‐to‐head [^123^I]ioflupane SPECT and [^18^F]AV133 PET): 68 individuals (sporadic PD, 51; hyposmia, 9; LRRK2, 2; healthy controls, 2; GBA, 1; PRKN, 1; RBD, 1; SWEDD, 1; sex, 43 males/25 females; age, 33–83 years; m, 64.0; sd, 9.1 years) were imaged with both [^123^I]ioflupane SPECT and [^18^F]AV133 PET. The interval between head‐to‐head scans was no more than 100 days and 45 subjects had multiple head‐to‐head acquisitions as part of a longitudinal assessment over a 2‐year period that led to a total of 162 paired scans.

**FIGURE 1:**
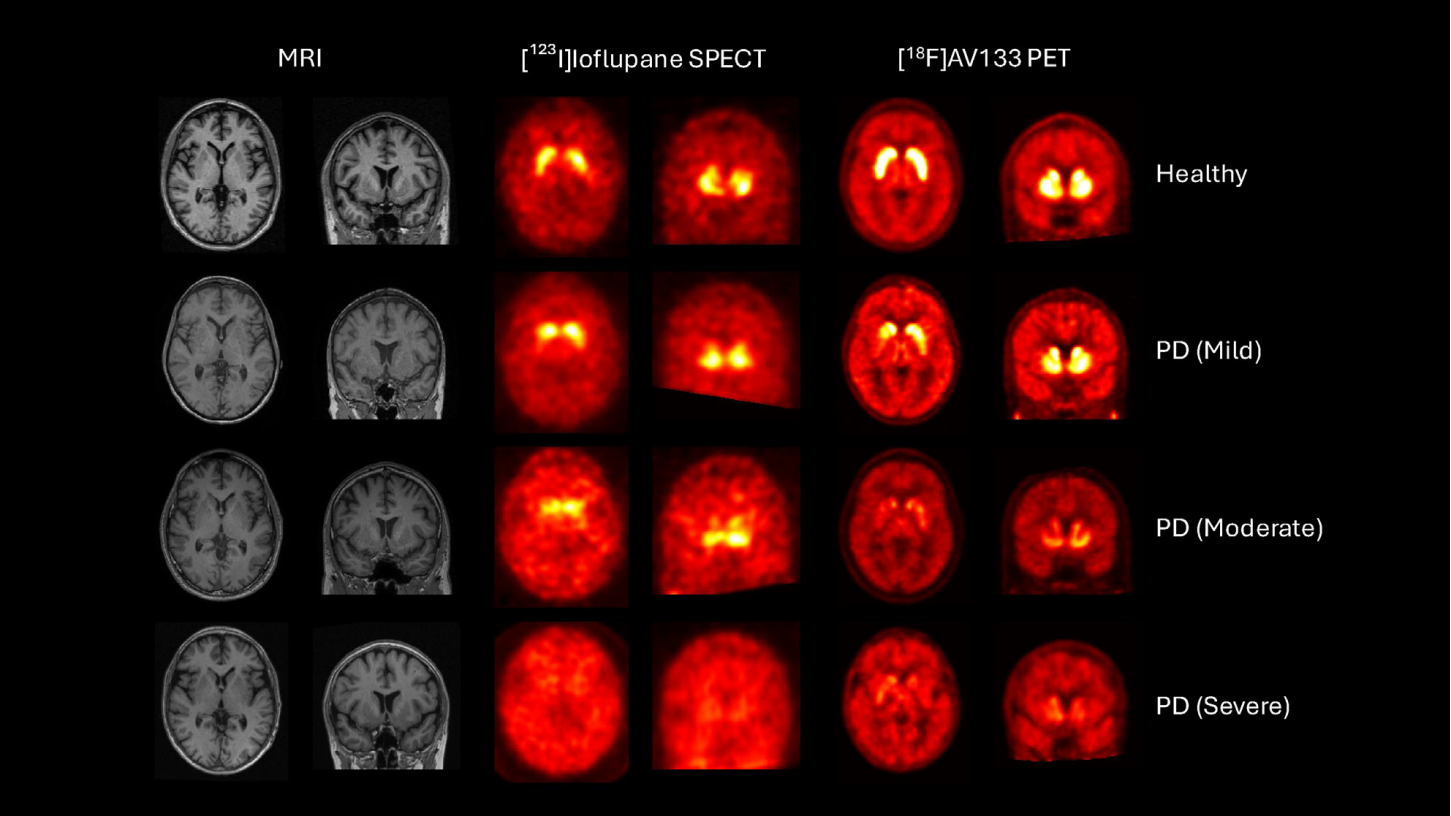
[^123^I]ioflupane single photon emission computed tomography (SPECT) (dopamine transporter [DAT]), [^18^F]AV133 positron emission tomography (PET) (VMAT2) and associated structural T1‐magnetic resonance imaging (MRI) images for a healthy subject, mild, moderate, and severe Parkinson's disease (PD) subjects. [Color figure can be viewed at www.annalsofneurology.org]

An example of [^123^I]ioflupane SPECT and [^18^F]AV133 PET imaging data obtained in the same subjects for 1 healthy and 3 PD subjects are shown in Figure 1, highlighting the differences in spatial resolution provided by the 2 modalities.

### Analysis Pipelines

**FIGURE 2:**
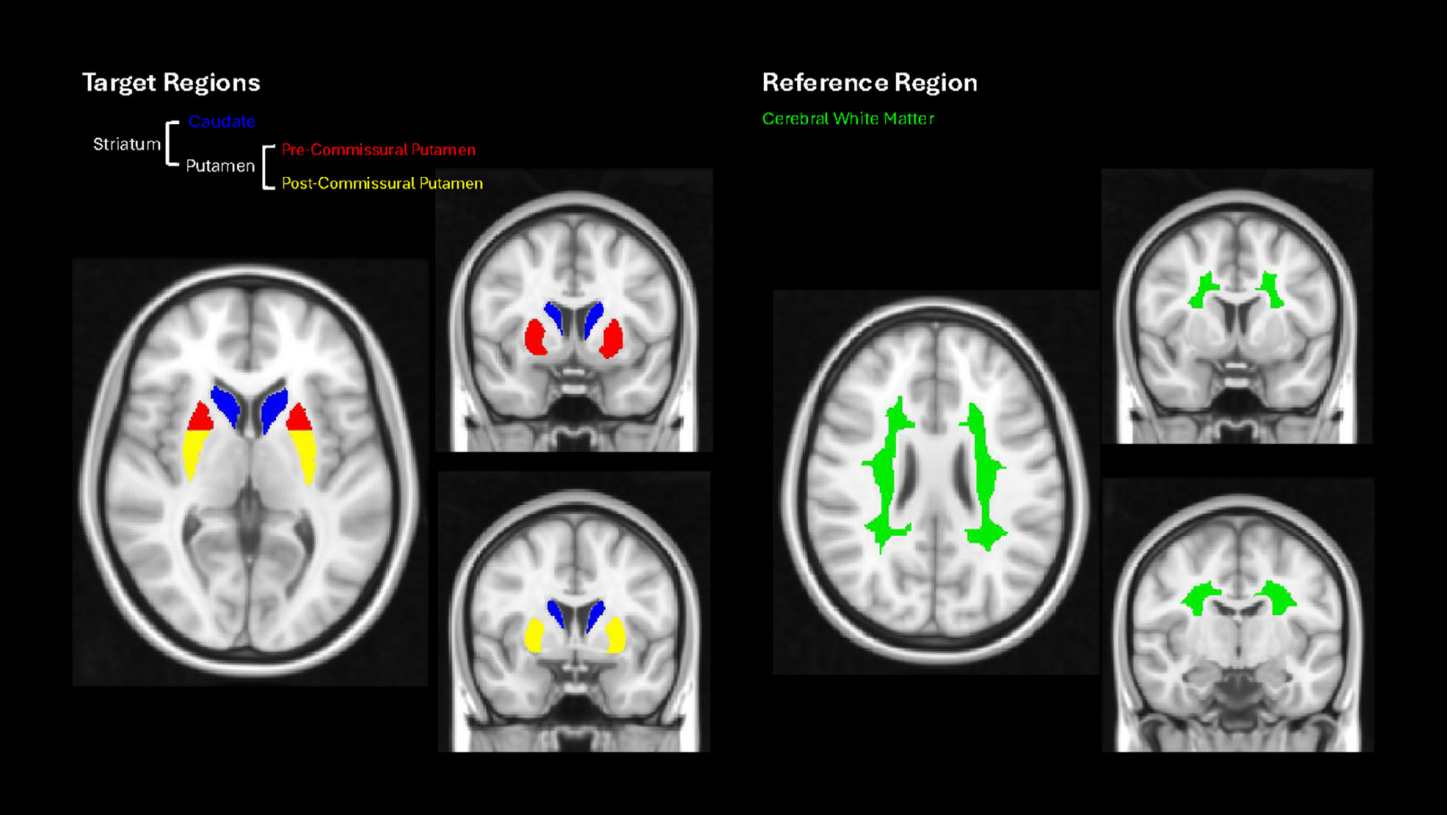
Target and reference regions defined in Montreal Neurological Institute (MNI152) space. [Color figure can be viewed at www.annalsofneurology.org]

All quantitative analyses were performed in MIAKAT (version 5.0).^22^ A standardized set of anatomical regions relevant to dopaminergic imaging defined in the CIC atlas^23^ were used (striatum, putamen, caudate, pre‐commissural putamen, post‐commissural putamen), and the reference region was chosen to be the cerebral white matter (CWM) because this has demonstrated increased performance over occipital reference region approaches.^14^ These target and reference regions are illustrated in Figure 2.

#### [ ^123^I]ioflupane SPECT Analysis Pipeline

SPECT imaging, such as [^123^I]ioflupane imaging, presents challenges in spatial registration and normalization because of the low resolution, which can impact the accuracy of signal quantification. Given that most SPECT studies acquire [^123^I]ioflupane scans without corresponding magnetic resonance imaging (MRI), the analysis pipeline developed aimed to maximize the quality of spatial normalization of the images in scenarios where only SPECT data was available.

A set of 17 SPECT templates, representing different stages of disease progression in PD, were generated in MNI152 space (Montreal Neurological Institute) to enable accurate normalization of the [^123^I]ioflupane SPECT data (see Figure S1). The 17 SPECT templates were derived from a healthy control SPECT template, with a range of progressively reduced signal intensities in the caudate and putamen, corresponding to increased deficits observed as PD progresses. The healthy control SPECT template was constructed as follows; a customized brain atlas (containing 23 regions of interest [ROIs]) specific to the dopamine transporter was first constructed in MNI152 space based on a sub‐sample of the CIC brain atlas (including left and right subregions of the basal ganglia, including the globus pallidus, ventral pallidum, pre‐caudate, post‐caudate, accumbens, pre‐dorsal putamen, pre‐ventral putamen, post‐dorsal putamen, and post‐ventral putamen, along with grey matter, white matter, cerebrospinal fluid (CSF), skull, and soft tissue.^23^ An optimization algorithm then determined the optimal atlas region values and 3 dimensional (3D) Gaussian smoothing kernel required to best match to a healthy [^123^I]ioflupane SPECT image template in MNI152 space leading to the estimate of 23 regional values and a smoothing kernel of 12mm full width at half maximum (FWHM). Subsequently, each voxel within the 23‐region brain atlas was populated with the associated regional estimates and convolved with the 12mm FWHM smoothing kernel to generate the primary healthy control SPECT template. This process was then repeated using the parameters given in Table 1 to generate the remaining 16 disease specific templates.

**TABLE 1:** Definition of the 17 [^123^I]ioflupane SPECT templates as a percentage of the HC values in the putamen and caudate

Template	Putamen‐L	Putamen‐R	Caudate‐L	Caudate‐R
1	100%	100%	100%	100%
2	75%	100%	100%	100%
3	50%	100%	100%	100%
4	25%	100%	100%	100%
5	100%	75%	100%	100%
6	100%	50%	100%	100%
7	100%	25%	100%	100%
8	0%	0%	100%	100%
9	0%	0%	75%	100%
10	0%	0%	50%	100%
11	0%	0%	25%	100%
12	0%	0%	100%	75%
13	0%	0%	100%	50%
14	0%	0%	100%	25%
15	0%	0%	75%	75%
16	0%	0%	50%	50%
17	0%	0%	25%	25%

All other regions contained 100% of the HC values.

HC = healthy controls; SPECT = single photon emission computed tomography.

Each [^123^I]ioflupane SPECT scan was spatially normalized into MNI152 space using an affine transformation to a linear combination (estimated as part of the normalization process) of the 17 SPECT templates. A weighted image was also applied as part of this process to prioritize signals from the basal ganglia region, thereby enhancing the alignment of the SPECT data in relation to this critical area of interest. QC was performed on all images to ensure the quality of spatial normalization.

#### [ ^18^F]AV133 PET Analysis Pipeline

T1‐MRI images were converted to 1mm isotropic format and brain segmentation into grey matter, white matter, and CSF was performed. DARTEL spatial normalization was then applied to derive mappings from the native space into MNI152 space using the standard T1 MNI152 template.^24^

For the [^18^F]AV133 PET images, motion correction was applied using a frame‐to‐frame rigid registration algorithm, with the first frame set as the default reference image. Following realignment, an average image was then generated. The average [^18^F]AV133 image was then rigidly registered to the T1‐MRI in native space and then spatially normalized into MNI152 space using the spatial transformation matrix derived previously. QC was performed on all images to ensure the quality of spatial normalization.

#### Calculation of Specific Binding Ratio Outcome Measures

The reference (REF) and target (TAR) ROIs from the CIC atlas were applied to the spatially normalized [^123^I]ioflupane SPECT and [^18^F]AV133 PET data in MNI152 space to derive the regional activity estimates SUV^REF^ and SUV^TAR^. This enabled quantification of regional specific binding ratio (SBR) values for the chosen target regions (left and right) using the white matter as the reference region.

(1) $${\mathrm{SBR}}^{\mathrm{TAR}}=\left({\mathrm{SUV}}^{\mathrm{TAR}}/{\mathrm{SUV}}^{\mathrm{REF}}\right)–1$$

### The Centamine Scale

The Centamines approach consists of 3 levels. The first level outlines the methodology used to define the Centamine scale using [^123^I]ioflupane SPECT data, ranging from a baseline of 100% representing healthy controls to 0% corresponding to an absence of specific binding. The second and third level analyses then detail how other tracers may be converted into the Centamine scale using head‐to‐head data sets with either [^123^I]ioflupane SPECT data or a different tracer that has been previously calibrated to the Centamine scale using a level 2 analysis.

#### Level 1: Specification of Centamines with [ ^123^I]ioflupane SPECT Data

The level 1 analysis defines the Centamine scale based on healthy [^123^I]ioflupane SPECT data.

#### Definition of Centamines

The Centamine scale provides a distinct Centamine value (CM) for a particular target ROI. To enable this, it is necessary to define the mean SBR value from the cohort of healthy controls (n = 227) for this ROI ($\mu \left({\mathrm{HC}\;\mathrm{SBR}}_{\mathrm{ROI}}^{\left[123I\right]\text{Ioflupane}}\right))$. The Centamine value of this ROI for an individual [^123^I]ioflupane scan (with an SBR value in that region of ${\mathrm{SBR}}_{\mathrm{ROI}}^{\left[123I\right]\text{Ioflupane}}$) can then be calculated as:

(2) $${\mathrm{CM}}_{\mathrm{ROI}}^{\left[123I\right]\text{Ioflupane}}=100\%\times \frac{{\mathrm{SBR}}_{\mathrm{ROI}}^{\left[123I\right]\text{Ioflupane}}}{\mu \left({\mathrm{HC}\;\mathrm{SBR}}_{\mathrm{ROI}}^{\left[123I\right]\text{Ioflupane}}\right)}.$$

#### Level 2: Mapping Tracer X into Centamines: Tracer X Versus [ ^123^I]Ioflupane SPECT

The level 2 analysis enables mapping of a different dopaminergic neuronal imaging marker, TracerX, into Centamines using a head‐to‐head data set acquired with [^123^I]ioflupane SPECT and TracerX in the same subjects. It is envisaged that such a data set would include approximately 50 subjects with a suitable range of dopamine neuron levels to allow accurate estimation of the mapping. This number aligns with the prior Centiloids work.^15^

A linear regression model is applied to the SBR head‐to‐head data from TracerX and $\left[123I\right]\text{Ioflupane}$ scans for a given ROI, to derive the slope $a_{\text{Tracer}\;X}$ and intercept $b_{\text{Tracer}\;X}$. This relationship is given by,

(3) $${\mathrm{SBR}}_{\mathrm{ROI}}^{\text{Tracer}\;X}=a_{\text{Tracer}\;X}\times {\mathrm{SBR}}_{\mathrm{ROI}}^{\left[123I\right]\text{Ioflupane}}+b_{\text{Tracer}\;X}.$$

The TracerXSBR values for CM = 0% and CM = 100% can then be determined for the given ROI by

(4) $${\mathrm{SBR}}_{\mathrm{ROI}}^{\text{Tracer}\;X\;\mathrm{CM}0}=a_{\text{Tracer}\;X}\times 0+b_{\text{Tracer}\;X}=b_{\text{Tracer}\;X},$$

(5) $${\mathrm{SBR}}_{\mathrm{ROI}}^{\text{Tracer}\;X\;\mathrm{CM}100}=a_{\text{Tracer}\;X}\times \mu \left({\mathrm{HC}\;\mathrm{SBR}}_{\mathrm{ROI}}^{\left[123I\right]\text{Ioflupane}}\right)+b_{\text{Tracer}\;X}.$$

The Centamine value for an individual TracerX scan can then be calculated as,

(6) $${\mathrm{CM}}_{\mathrm{ROI}}^{\text{Tracer}\;X}=100\%\times \frac{{\mathrm{SBR}}_{\mathrm{ROI}}^{\text{Tracer}\;X}-{\mathrm{SBR}}_{\mathrm{ROI}}^{\text{Tracer}\;X\;\mathrm{CM}0}}{{\mathrm{SBR}}_{\mathrm{ROI}}^{\text{Tracer}\;X\;\mathrm{CM}100}-{\mathrm{SBR}}_{\mathrm{ROI}}^{\text{Tracer}\;X\;\mathrm{CM}0}\;},$$

where ${\mathrm{SBR}}_{\mathrm{ROI}}^{\text{Tracer}\;X}$ denotes SBR values for the individual TracerX scan. By substituting the values for ${\mathrm{SBR}}_{\mathrm{ROI}}^{\text{Tracer}\;X\;\mathrm{CM}0}$ and ${\mathrm{SBR}}_{\mathrm{ROI}}^{\text{Tracer}\;X\;\mathrm{CM}100}$ from Equations (4) and (5), the final form of the Centamine equation for TracerX is,

(7) $${\mathrm{CM}}_{\mathrm{ROI}}^{\text{Tracer}\;X}=100\%\times \frac{{\mathrm{SBR}}_{\mathrm{ROI}}^{\text{Tracer}\;X}-b_{\text{Tracer}\;X}}{a_{\text{Tracer}\;X}\times \mu \left(\mathrm{HC}\;{\mathrm{SBR}}_{\mathrm{ROI}}^{\left[123I\right]\text{Ioflupane}}\right)}.$$

Mapping [^18^F]AV133 PET into Centamines.

Using this framework, a level 2 analysis was performed with the [^18^F]AV133 PET versus [^123^I]ioflupane SPECT Head‐to‐Head data set involving 68 subjects with a total of 162 paired scans (data set 2). In this analysis, all the paired available scans were treated as independent data points.

#### Level 3: Mapping Tracer Y into Centamines: Tracer Y Versus Tracer X

The level 3 analysis enables mapping of a different dopaminergic neuronal imaging marker, TracerY, into Centamines using a head‐to‐head data set acquired with TracerY and TracerX in the same subjects. This is conditional on TracerX having previously undergone a level 2 analysis using a TracerX versus $\left[123I\right]\text{ioflupane}\;\text{SPECT}$ head‐to‐head data set. Again, it is envisaged that such a data set would include approximately 50 subjects with a suitable range of dopamine neuron levels to allow accurate estimation of the mapping.

A linear regression model is applied to the SBR head‐to‐head data from TracerY and TracerX scans for a given ROI, to derive the slope $a_{\text{Tracer}\;Y}$ and intercept $b_{\text{Tracer}\;Y}$. This relationship is given by,

(8) $${\mathrm{SBR}}_{\mathrm{ROI}}^{\text{Tracer}\;Y}=a_{\text{Tracer}\;Y}\times {\mathrm{SBR}}_{\mathrm{ROI}}^{\text{Tracer}\;X}+b_{\text{Tracer}\;Y}.$$

Using known TracerX values for CM = 0% and CM = 100% derived from the level 2 analysis, the corresponding TracerYSBR values for CM = 0% and CM = 100% can be determined for the given ROI as,

(9) $${\mathrm{SBR}}_{\mathrm{ROI}}^{\text{Tracer}\;Y\;\mathrm{CM}0}=a_{\text{Tracer}\;Y}\times {\mathrm{SBR}}_{\mathrm{ROI}}^{\text{Tracer}\;X\;\mathrm{CM}0}+b_{\text{Tracer}\;Y},$$

(10) $${\mathrm{SBR}}_{\mathrm{ROI}}^{\text{Tracer}\;Y\;\mathrm{CM}100}=a_{\text{Tracer}\;Y}\times {\mathrm{SBR}}_{\mathrm{ROI}}^{\text{Tracer}\;X\;\mathrm{CM}100}+b_{\text{Tracer}\;Y}.$$

The Centamine value for an individual TracerY scan is then be calculated by,

(11) $${\mathrm{CM}}_{\mathrm{ROI}}^{\text{Tracer}\;Y}=100\%\times \frac{{\mathrm{SBR}}_{\mathrm{ROI}}^{\text{Tracer}\;Y}-{\mathrm{SBR}}_{\mathrm{ROI}}^{\text{Tracer}\;Y\;\mathrm{CM}0}}{{\mathrm{SBR}}_{\mathrm{ROI}}^{\text{Tracer}\;Y\;\mathrm{CM}100}-{\mathrm{SBR}}_{\mathrm{ROI}}^{\text{Tracer}\;Y\;\mathrm{CM}0}\;},$$

where ${\mathrm{SBR}}_{\mathrm{ROI}}^{\text{Tracer}\;Y}$ denotes SBR values for the individual TracerY scan. By substituting the values for ${\mathrm{SBR}}_{\mathrm{ROI}}^{\text{Tracer}\;Y\;\mathrm{CM}0}$, ${\mathrm{SBR}}_{\mathrm{ROI}}^{\text{TracerY}\;\mathrm{CM}100}$, ${\mathrm{SBR}}_{\mathrm{ROI}}^{\text{Tracer}\;X\;\mathrm{CM}0}$ and ${\mathrm{SBR}}_{\mathrm{ROI}}^{\text{Tracer}\;X\;\mathrm{CM}100}$ from Equations (4), (5), (9), and (10), the final form of the Centamine equation for TracerY is

(12) $$\begin{array}{l} {\mathrm{CM}}_{\mathrm{ROI}}^{\text{Tracer}\;Y}=100\%\\ \;\times \frac{{\mathrm{SBR}}_{\mathrm{ROI}}^{\text{Tracer}\;Y}-a_{\text{Tracer}\;Y}\times b_{\text{Tracer}\;X}-b_{\text{Tracer}\;Y}}{a_{\text{Tracer}\;Y}\times (a_{\text{Tracer}\;X}\times \mu ({\mathrm{HC}\;\mathrm{SBR}}_{\mathrm{ROI}}^{\left[123I\right]\text{Ioflupane}}))}. \end{array}$$

### Correction for Age and Sex Effects

Prior harmonization approaches, such as Centiloids^15^ and Centaurs,^19^ have not corrected their outcome measures for age and sex effects, and we align with those approaches here, in terms of the definition of CM. However, in applications like stratification or diagnostics, especially those focussing on the putamen, it may be desirable to explicitly correct for these effects because dopaminergic neuronal density measured by DAT imaging shows a modest age‐related decline (approx. 5% per decade)^17^, ^25^, ^26^; and sex differences (females approx. 10–15% higher).^26^ To this end, we propose an example of age and sex corrected Centamine (${\mathrm{CM}}^{*}$) outcome measure that may be further helpful in these applications. This transformation is determined from the level 1 output data with DAT in Centamines from healthy controls. A linear regression is performed on these data using the following equation,

(13) $${\mathrm{CM}}_{\mathrm{ROI}}^{\mathrm{HC}}\left(\mathrm{age},\mathrm{sex}\right)=a^{\mathrm{HC}}\times \mathrm{age}+b^{\mathrm{HC}}\times \mathrm{sex}+c^{\mathrm{HC}},$$

where ${\mathrm{CM}}_{\mathrm{ROI}}$ is the Centamine value in ROI, age is the age at the time of the scan and sex is 0 for female and 1 for male subjects. We propose to anchor ${\mathrm{CM}}^{*}$ to the expected average CM value for a 65‐year‐old person, which yields the following equation,

(14) $${\mathrm{CM}}_{\mathrm{ROI}}^{*}=\frac{{\mathrm{CM}}_{\mathrm{ROI}}^{\mathrm{HC}65}\left(65,\;0.5\right)\;}{{\mathrm{CM}}_{\mathrm{ROI}}^{\mathrm{HC}}\left(\mathrm{age},\mathrm{sex}\right)\;}{\mathrm{CM}}_{\mathrm{ROI}},$$

or explicitly,

(15) $${\mathrm{CM}}_{\mathrm{ROI}}^{*}=\frac{a^{\mathrm{HC}}\times 65+b^{\mathrm{HC}}\times 0.5+c^{\mathrm{HC}}\;}{a^{\mathrm{HC}}\times \mathrm{age}+b^{\mathrm{HC}}\times \mathrm{sex}+c^{\mathrm{HC}}}{\mathrm{CM}}_{\mathrm{ROI}},$$

where age and sex are the actual age and sex of the subject whose Centamine value is ${\mathrm{CM}}_{\mathrm{ROI}}.$

## Results

All [^123^I]ioflupane SPECT and [^18^F]AV133 scans were successfully spatially normalized into MNI152 space, as assessed by visual assessment, and SBR values were calculated for left and right striatum, putamen, caudate, pre‐commissural putamen and post‐commissural putamen.

### Level 1 Analysis: [ ^123^I]ioflupane SPECT

The results from the level 1 analysis of the [^123^I]ioflupane SPECT data to derive the SBR anchor points for Centamine values of 0 and 100% for [^123^I]ioflupane SPECT are provided in Table 2.

**TABLE 2:** Results of the level 1 and level 2 analyses detailing the SBR anchor points of CM0 and CM100 for [^123^I]ioflupane SPECT and [^18^F]AV133 PET for the 5 target ROIs

	Level 1 analysis ([^123^I]ioflupane SPECT)		Level 2 analysis ([^18^F]AV133 PET vs [^123^I]ioflupane SPECT)						
	[^123^I]ioflupane SPECT		${\mathrm{SBR}}_{\mathrm{ROI}}^{\left[18F\right]\mathrm{AV}133}=a_{\left[18F\right]\mathrm{AV}133}\times {\mathrm{SBR}}_{\mathrm{ROI}}^{\left[123I\right]\text{Ioflupane}}+b_{\left[18F\right]\mathrm{AV}133}$					[^18^F]AV133 PET	
ROI	CM0 (SBR)	CM100 (SBR)	$a_{\left[18F\right]\mathrm{AV}133}$	95% CI	$b_{\left[18F\right]\mathrm{AV}133}$	95% CI	*R* ^*2*^	CM0 (SBR)	CM100 (SBR)
Striatum	0	1.41	1.48	1.28, 1.69	0.81	0.63, 0.99	61%	0.81	2.90
Putamen	0	1.56	1.93	1.72, 2.13	0.64	0.47, 0.81	73%	0.64	3.65
Caudate	0	1.23	1.14	0.94, 1.34	0.79	0.63, 0.96	51%	0.79	2.19
Pre‐commissural putamen	0	1.86	1.36	1.14, 1.59	0.98	0.72, 1.23	52%	0.98	3.51
Post‐commissural putamen	0	1.33	2.38	2.20, 2.57	0.59	0.47, 0.71	83%	0.59	3.75

CI = confidence interval; CM = Centamines; CM0 = CM = 0%; CM100 = CM = 100%; PET = positron emission tomography; ROI, region of interest; SBR = specific binding ratio; SPECT = single photon emission computed tomography.

### Level 2 Analysis: [ ^18^F]AV133 PET Versus [ ^123^I]ioflupane SPECT

**FIGURE 3:**
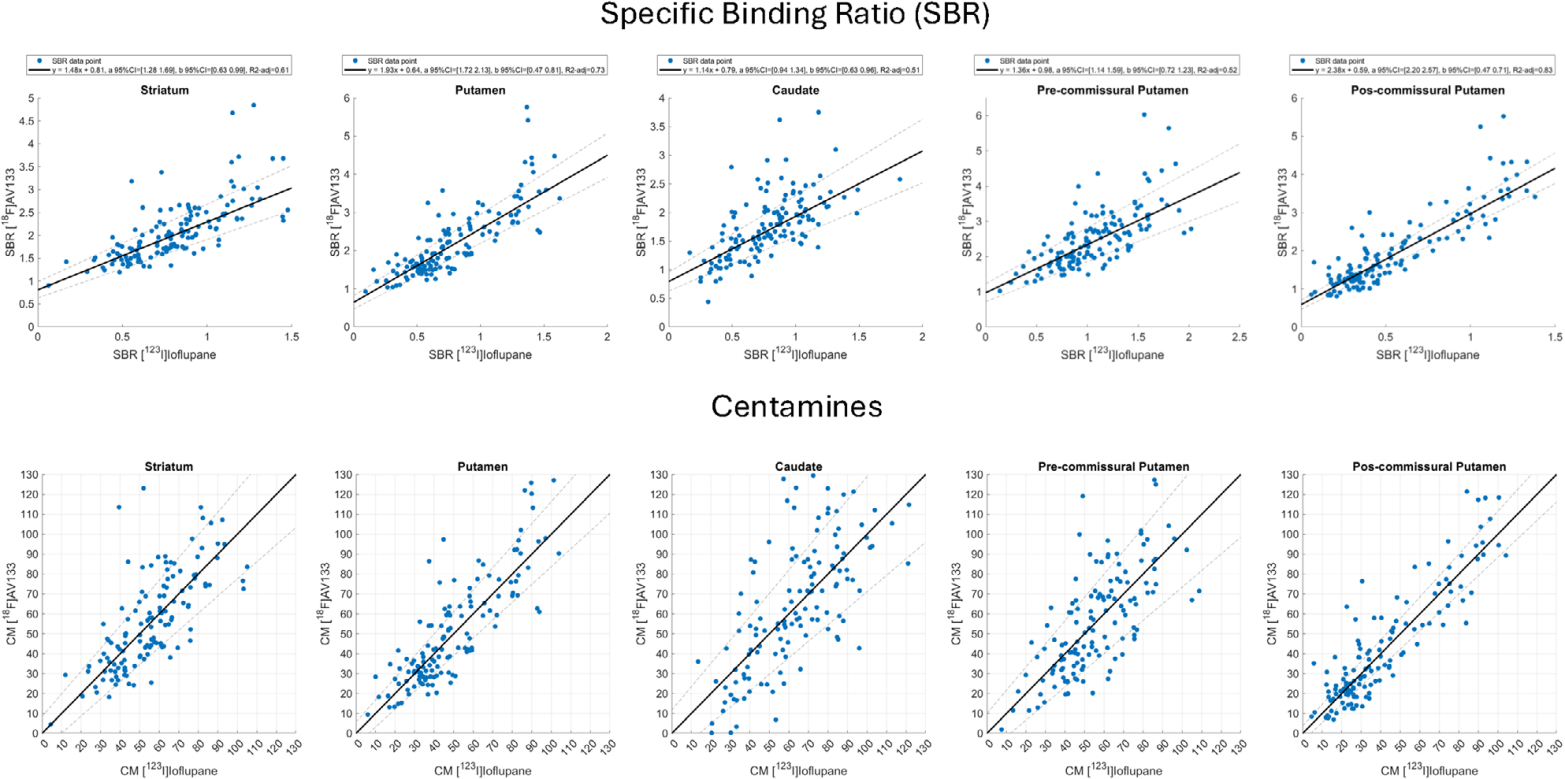
Results from the level 2 regression analyses of the head‐to‐head [^18^F]AV133 positron emission tomography (PET) and [^123^I]ioflupane single photon emission computed tomography (SPECT) specific binding ratio (SBR) data (top row) and transformation of the data into the Centamine scale (bottom row) for the 5 target regions of interest. Note that a small number of points are outside the axis range of 0–130 in the Centamine plots and are not displayed. [Color figure can be viewed at www.annalsofneurology.org]

The level 2 analysis was performed using the baseline data from data set 2. The results from the level 2 analysis of the [^18^F]AV133 PET and [^123^I]ioflupane SPECT to derive the SBR anchor points for Centamine values of 0 and 100% for [^18^F]AV133 PET are provided in Table 2. The linear regressions for the 5 target regions are displayed in Figure 3 along with their subsequent transformation into Centamines, demonstrating a moderate‐strong relationship between [^18^F]AV133 PET and [^123^I]ioflupane SPECT. The calculated regional *R* ^2^ ranged from 51 to 83%, with the highest value observed in the post‐commissural putamen and the lowest value in the caudate. The highest slope and lowest intercept were both obtained in the post‐commissural putamen. Overall, the most important regions for PD research applications (post‐commissural putamen, putamen, and striatum) provided the best agreements. Additional information on the analysis of the agreement between the Centamine values are provided in Figures S2–S4, which include Bland–Altman plots, histograms of the Centamine values and the individual differences across [^18^F]AV133 PET and [^123^I]ioflupane SPECT.

**FIGURE 4:**
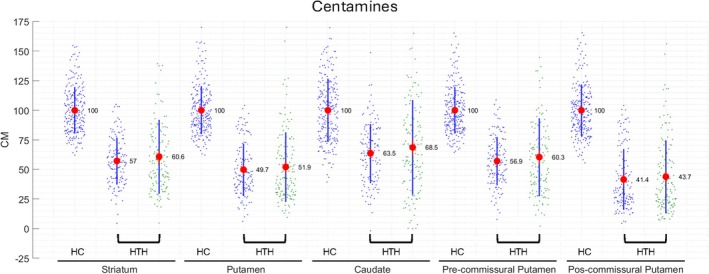
Data from data sets 1 and 2 mapped into the Centamine scale following level 1 and 2 analyses (data set 1 [HC], data set 2 [HTH], [^123^I]ioflupane single photon emission computed tomography (SPECT) (blue), [^18^F]AV133 positron emission tomography (PET) (green), group means displayed as red dots and black lines indicate ±SD. HC = healthy controls, HTH = head‐to‐head. [Color figure can be viewed at www.annalsofneurology.org]

Figure 4 illustrates the data mapped into Centamines and confirms a good alignment of the head‐to‐head [^18^F]AV133 PET and [^123^I]ioflupane SPECT data across all 5 regions with a minimum group‐level difference in putamen (2.2%) and a maximum group difference in caudate (5%). The head‐to‐head data demonstrated an increasing reduction in Centamines from caudate (lowest reduction), through striatum, pre‐commissural putamen and putamen to post‐commissural putamen (highest reduction) consistent with the prevalence of PD subjects in this cohort and the known loss of dopaminergic neurons because of the disease.

Analysis of the full longitudinal data set yielded results consistent with the baseline data, although correlations were on average approximately 6% lower (see Figures S5 and S6). The longitudinal data also enabled a longitudinal analysis to calculate the mean change per annum, using linear regression analysis, for each imaging marker following Centamine transformation. The results for the mean change are given in Table 3 and demonstrate that this was similar for both imaging markers.

**TABLE 3:** Longitudinal analysis of the head‐to‐head data transformed into Centamines to calculate mean changes per annum

Region	% CM change per annum		Absolute CM change per annum	
	[^123^I]ioflupane SPECT	[^18^F]AV133 PET	[^123^I]ioflupane SPECT	[^18^F]AV133 PET
Striatum	−6.4	−7.4	−3.3	−3.7
Putamen	−7.1	−7.2	−3.1	−3.0
Caudate	−6.8	−6.7	−3.7	−4.6
Pre‐commissural putamen	−7.1	−8.2	−3.8	−3.6
Post‐commissural putamen	−7.3	−7.4	−2.3	−2.6

CM = Centamines; PET = positron emission tomography; SPECT = single photon emission computed tomography.

### Correction for Age and Sex Effects

**FIGURE 5:**
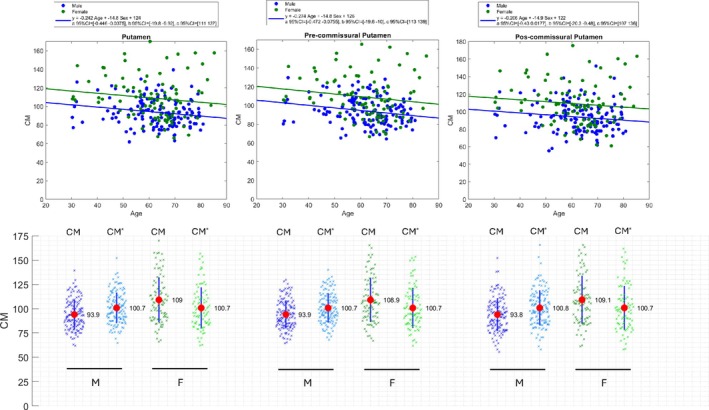
Linear regression analysis of the healthy [^123^I]ioflupane single photon emission computed tomography (SPECT) data with age and sex as regressors for putamen regions (top) and Centamine (CM) and age/sex corrected Centamine (${\mathrm{CM}}^{*}$) values for male (M) and female (F) groups (bottom). [Color figure can be viewed at www.annalsofneurology.org]

Linear regression analyses were performed on the Centamine values derived from the healthy [^123^I]ioflupane SPECT data (n = 227) for putamen regions with age and sex as regressors using Equation (13) (Figure 5). This, then, enabled calculation of the age and sex corrected Centamine values (${\mathrm{CM}}^{*}$) using Equation (15). The putamen demonstrated a 14.8% higher Centamine values in females and a reduction of approximately 2.4% per decade in both sexes. Similar results were obtained for the subregions of the putamen with slightly higher age‐related decline in the pre‐commissural putamen (2.7%/decade) as compared to the post‐commissural putamen (2.1%/decade). The comparison of the group distributions for ${\mathrm{CM}}^{*}$ versus CM can be seen in Figure 5 where it is evident that there is a modest reduction in variance and alignment of means with 100% for both male and females. Although modest, this correction will likely be important for stratification/diagnostic applications using a cut‐off value to determine a classification of dopamine neuron deficit.

To further assess the performance of ${\mathrm{CM}}^{*}$ in classifying dopaminergic neuronal deficits, a threshold of 75% for the lowest‐sided putamen was applied to the head‐to‐head [^123^I]ioflupane SPECT and [^18^F]AV133 PET data. Using this criterion, [^123^I]ioflupane SPECT and [^18^F]AV133 PET classified 86% and 84% of subjects, respectively, as having low dopaminergic neuron levels, with >95% concordance (Cohen's κ = 0.81), indicating excellent inter‐modality agreement.

## Discussion

The Centamine scale has been proposed to enable a harmonized quantification framework for dopaminergic imaging with molecular imaging tracers. The Centamine methodology will enable multiple dopamine neuron imaging biomarkers to be compared and even for multiple imaging biomarkers to be used in a single clinical study potentially informing and accelerating PD clinical trials throughout the course of disease. This approach builds on the success of Centiloids and Centaurs, which have proposed and developed harmonized amyloid‐ and tau‐PET quantification in AD, simplifying eligibility assessment, enhancing longitudinal data reporting and improving cross‐tracer comparability.

Developing the Centamine scale is particularly timely given the recent paradigm shift to a biologic definition of PD and related synucleinopathies and the integrated biologic and clinical NSD‐ISS.^2^ Dopamine imaging is a key anchor biomarker for NSD staging. Understanding the earliest stages of dopamine dysfunction may enable studies investigating molecular determinants of neurodegeneration as disease progresses. The availability of a standardized quantitative dopamine imaging outcome would provide an intuitive scale to enable clear and reliable disease staging for research studies. The Centamine strategy would also accelerate therapeutic studies by enabling multiple tracers to be used in a single study. Centamines would further enable comparison of dopaminergic neuronal loss in different brain regions that might inform disease progression throughout the disease both before typical symptoms and after functional impairment is present. Finally, Centamines would also allow an easier comparison between clinical therapeutic trials and potentially between different clinical syndromes, for example, PD and multiple system atrophy.

The Centamine approach follows a similar principle to Centiloids and Centaurs, but with key differences. Unlike amyloid and tau, which accumulate over time with disease progression, dopaminergic neuron levels decline from a normal baseline. As a result, the Centamine scale is inverted, with 100% representing a healthy state, whereas in Centiloids and Centaurs, 100% corresponds to the average amyloid‐ and tau‐PET signal in typical AD. This inversion somewhat simplifies the definition of the Centamine scale, as it can be directly established from a healthy population without needing to anchor 100% to a specific disease stage, as required by Centiloids and Centaurs. Accordingly, a Centamine value of 100% denotes the mean specific binding observed in healthy controls, whereas a value of 0% represents complete loss of dopaminergic neurons, corresponding to the absence of a specific binding signal. It should be noted that the CM* scale for [^123^I]ioflupane SPECT is related to the percent deviation reported by DaTQUANT software (GE Healthcare Limited, Chalfont St Giles, UK)^27^, ^28^, which instead anchors the mean of (age‐dependent) normal to be 0 and the absence of signal (SBR = 0) to be 1.

Furthermore, Centamine values are by necessity region‐specific, given the heterogeneous and region‐specific loss of dopamine neuron levels in PD. This allows for multiple Centamine values per scan, making it functionally closer to Centaurs, which also accounts for regional variation, rather than Centiloids, which assigns a single value per scan because of the uniform accumulation rate of amyloid across the brain.^29^ This offers another opportunity to use Centamines to investigate differences in regional pathology in PD and related synucleinopathies.

The Centamine framework consists of 3 levels of analysis to standardize the quantification of dopaminergic imaging tracers. This study presents a level 1 analysis, which defines the Centamine scale using a large cohort of healthy individuals (n = 227) imaged with [^123^I]ioflupane SPECT. A level 2 analysis was conducted using head‐to‐head imaging with [^18^F]AV133 PET and [^123^I]ioflupane SPECT, enabling the additional conversion of [^18^F]AV133 PET into the Centamine scale. The framework also outlines a level 3 analysis, which facilitates the conversion of additional tracers without requiring a direct head‐to‐head [^123^I]ioflupane SPECT data set. Instead, it leverages a head‐to‐head comparison with another tracer that has already undergone a level 2 analysis, ensuring continuity in tracer standardization.

The framework enables the harmonization of additional dopaminergic imaging markers, with ongoing head‐to‐head studies aimed at further expanding the Centamine scale. Current studies include [^18^F]PE2I PET versus [^123^I]ioflupane SPECT ([^18^F]PE2I PET through a level 2 analysis) and [^18^F]FPCIT PET versus [^18^F]AV133 PET ([^18^F]FPCIT through a level 3 analysis). These data sets will support the future integration of these tracers into the Centamine scale, increase our understanding of individual tracer signal‐to‐noise and expand its applicability.

As with many studies, there are certain limitations. In the level 2 analysis of [^18^F]AV133 PET and [^123^I]ioflupane SPECT R^2^ values ranged from 51 to 83% for the 5 regions considered with the putamen regions showing the highest correspondence (putamen = 73% and post‐commissural putamen = 83%). These correlations are somewhat lower than those typically reported for Centiloid analyses, particularly in the non‐putamen regions. Several factors likely contribute to this difference; (1) Centiloids only uses PET as an imaging modality. Here, we are using SPECT for DAT measurements, which is a modality that has a higher variability, (2) related to this is that PET processing benefits from the use of an associated structural T1‐MRI data for spatial normalization given its higher resolution/sensitivity enabling more accurate delineation of ROIs; (3) Centiloids derives its estimates from a much larger ROI (whole cortical region), so signal to noise would be higher and consequently the correlation would be expected to be higher too; and (4) in the Centiloids work there is often a significant cluster of healthy controls centered around the origin that helps boost the correlation a bit.

Taken together, these factors suggest that the most robust mappings and highest inter‐modality alignment are achieved in the putamen regions—those most relevant for PD imaging applications.

In theory, if 2 molecular imaging markers measure the same target in the same person, their SBR values may be expected to follow a simple mathematical relationship: *y = ax*. However, in this study, an intercept term was needed to accurately describe the relationship between [^18^F]AV133 PET and [^123^I]ioflupane SPECT. This intercept was fairly consistent across the 5 brain regions analyzed (0.60–0.86) (see Table 1). It may be because of factors like differences in non‐specific binding between target and reference regions, limitations in [^123^I]ioflupane SPECT's sensitivity at low dopamine levels, or variations in DAT and VMAT2 expression as the disease progresses. The need for an intercept is not surprising, as similar findings have been seen in studies using Centiloids and Centaurs. The future analysis of the [^18^F]PE2I PET versus [^123^I]ioflupane SPECT and [^18^F]FPCIT PET versus [^18^F]AV133 PET data sets will provide further insights into these relationships. In summary, this study confirms a significant relationship between [^18^F]AV133 PET and [^123^I]ioflupane SPECT, with the Centamine scale providing a useful way to align them.

Although the Centamine method offers a standardized framework for comparing results across different dopaminergic imaging tracers, additional variability may arise from differences in scanner hardware, image reconstruction algorithms, and acquisition protocols. The use of standardized acquisition windows is essential to minimize bias related to non–steady‐state imaging. Further work is warranted to systematically assess the impact of scanner‐ and reconstruction‐related factors on potential sources of additional bias.

The proposed approach is designed to allow individual research groups to calibrate their own image analysis pipelines, ensuring they can generate results in Centamines. To enable this, the requisite healthy [^123^I]ioflupane SPECT and head‐to‐head data sets will be made available (see http://ppmi-info.org for details). To calibrate an image analysis pipeline to output values in Centamines, a lab would need to process imaging data with the standard TAR and REF ROIs, all of which are provided on the PPMI website (http://ppmi-info.org). The process required is as follows:

- Calibration of [^123^I]ioflupane SPECT pipeline into Centamines (level 1 analysis): the independent lab would process all of the imaging data in data set 1 (healthy [^123^I]ioflupane SPECT) to derive regional SBR values from their own pipeline. Next, in conjunction with the Centamine values that we have provided for the scans in data set 1, a linear regression can be applied, by region, to derive the regional Centamine mappings for the new analysis pipeline.

$${\mathrm{CM}}_{\mathrm{ROI}}^{\left[123I\right]\text{Ioflupane}}=a_{\text{NewPipeline}}\times {\mathrm{SBR}}_{\mathrm{ROI}}^{\left[123I\right]\text{Ioflupane}}+b_{\text{NewPipeline}}$$

- Calibration of Tracer X pipeline into Centamines (level 2 analysis): the independent lab would process all of the imaging data from a head‐to‐head [^123^I]ioflupane SPECT and Tracer X data set (eg, head‐to‐head [^123^I]ioflupane SPECT and [^18^F]AV133 PET) to derive regional SBR values for Tracer X and Centamine values for [^123^I]ioflupane SPECT (using the prior level 1 calibration) from their own pipeline. Next, in conjunction with the Centamine values that we have provided for the scans in data set 2, a linear regression can be applied, by region, with the SBR data from Tracer X to derive the regional Centamine mappings for the new analysis pipeline.

$${\mathrm{CM}}_{\mathrm{ROI}}^{\text{Tracer}\;X}=c_{\text{NewPipeline}}\times {\mathrm{SBR}}_{\mathrm{ROI}}^{\text{Tracer}\;X}+d_{\text{NewPipeline}}$$

In addition, the lab should confirm alignment of the derived [^123^I]ioflupane SPECT Centamine values with those provided.

- Calibration of Tracer Y pipeline into Centamines (level 3 analysis): the independent lab would process all of the imaging data from a head‐to‐head Tracer X and Tracer Y data set to derive regional SBR values for Tracer Y and Centamine values for Tracer X (using the prior level 2 calibration) from their own pipeline. Next, in conjunction with the Centamine values, that will be provided in the future for Tracer Y versus Tracer X data sets, a linear regression can be applied, by region, with the SBR data from Tracer Y to derive the regional Centamine mappings for the new analysis pipeline.

$${\mathrm{CM}}_{\mathrm{ROI}}^{\text{Tracer}\;Y}=e_{\text{NewPipeline}}\times {\mathrm{SBR}}_{\mathrm{ROI}}^{\text{Tracer}\;Y}+f_{\text{NewPipeline}}$$

In addition, the lab should confirm alignment of the derived Tracer X Centamine values with those provided (Table 2).

We have successfully developed the Centamine scale for harmonized quantification of dopaminergic neuronal imaging markers in PD and related synucleinopathies with the objective of further enabling and accelerating clinical therapeutic trials and diagnostic applications.

## Author Contributions

Z.F., J.E., K.M., J.S., and R.G. contributed to the conception and design of the study; Z.F., G.S., G.R., J.A., K.M., J.S., and R.G. contributed to the acquisition and analysis of data; Z.F., G.S., G.R., J.A., P.C., R.C., G.K., L.P., C.S., A.S., L.I., G.T., J.E., K.M., J.S., and R.G. contributed to drafting the text or preparing the figures.

## Potential Conflicts of Interest

R.G. and G.S. are founders and directors of MIAKAT. The remaining authors have nothing to report.

## PPMI STUDY TEAMS/CORES/COLLABORATORS FOR PUBLICATIONS

**Executive Steering Committee**:

Kenneth Marek, MD^1^ (Principal Investigator); Caroline Tanner, MD, PhD^9^; Tanya Simuni, MD^3^; Andrew Siderowf, MD, MSCE^12^; Douglas Galasko, MD^27^; Lana Chahine, MD^41^; Christopher Coffey, PhD^4^; Kalpana Merchant, PhD^61^; Kathleen Poston, MD^40^; Roseanne Dobkin, PhD^43^; Tatiana Foroud, PhD^15^; Brit Mollenhauer, MD^8^; Dan Weintraub, MD^12^; Ethan Brown, MD^9^; Karl Kieburtz, MD, MPH^23^; Mark Frasier, PhD^6^; Todd Sherer, PhD^6^; Sohini Chowdhury, MA^6^; Roy Alcalay, MD^36^ and Aleksandar Videnovic, MD^47^

**Steering Committee**:

Duygu Tosun‐Turgut, PhD^9^; Werner Poewe, MD^7^; Susan Bressman, MD^14^; Jan Hammer^15^; Raymond James, RN^22^; Ekemini Riley, PhD^42^; John Seibyl, MD^1^; Leslie Shaw, PhD^12^; David Standaert, MD, PhD^18^; Sneha Mantri, MD, MS^62^; Nabila Dahodwala, MD^12^; Michael Schwarzschild^47^; Connie Marras^45^; Hubert Fernandez, MD^25^; Ira Shoulson, MD^23^; Helen Rowbotham^2^; Paola Casalin^11^ and Claudia Trenkwalder, MD^8^

**Michael J. Fox Foundation (Sponsor)**:

Todd Sherer, PhD; Sohini Chowdhury, MA; Mark Frasier, PhD; Jamie Eberling, PhD; Katie Kopil, PhD; Alyssa O'Grady; Maggie McGuire Kuhl; Leslie Kirsch, EdD and Tawny Willson, MBS

**Study Cores, Committees and Related Studies**:

*Project Management Core*: Emily Flagg, BA1

*Site Management Core*: Tanya Simuni, MD3; Bridget McMahon, BS1

Strategy and Technical Operations: Craig Stanley, PhD1; Kim Fabrizio, BA1

*Data Management Core*: Dixie Ecklund, MBA, MSN4; Trevis Huff, BSE4

*Screening Core*: Tatiana Foroud, PhD15; Laura Heathers, BA15; Christopher Hobbick, BSCE15; Gena Antonopoulos, BSN15

*Imaging Core*: John Seibyl, MD1; Kathleen Poston, MD40

*Statistics Core*: Christopher Coffey, PhD4; Chelsea Caspell‐Garcia, MS4; Michael Brumm, MS4

*Bioinformatics Core*: Arthur Toga, PhD10; Karen Crawford, MLIS10 *Biorepository Core*: Tatiana Foroud, PhD15; Jan Hamer, BS15

*Biologics Review Committee*: Brit Mollenhauer8; Doug Galasko27; Kalpana Merchant61

*Genetics Core*: Andrew Singleton, PhD13

*Pathology Core*: Tatiana Foroud, PhD15; Thomas Montine, MD, PhD40

*Found*: Caroline Tanner, MD PhD9

*PPMI Online*: Carlie Tanner, MD PhD9; Ethan Brown, MD9; Lana Chahine, MD41; Roseann Dobkin, PhD43; Monica Korell, MPH9

**Site Investigators**:

Charles Adler, PhD^51^; Roy Alcalay, MD^36^; Amy Amara, PhD^52^; Paolo Barone, PhD^30^; Bastiaan Bloem, PhD^60^ Susan Bressman, MD^14^; Kathrin Brockmann, MD^26^; Norbert Brüggemann, MD^59^; Lana Chahine, MD^41^; Kelvin Chou, MD^44^; Nabila Dahodwala, MD^12^; Alberto Espay, MD^32^; Stewart Factor, DO^16^; Hubert Fernandez, MD^25^; Michelle Fullard, MD^52^; Douglas Galasko, MD^27^; Robert Hauser, MD^19^; Penelope Hogarth, MD^17^; Shu‐Ching Hu, PhD^21^; Michele Hu, PhD^58^; Stuart Isaacson, MD^31^; Christine Klein, MD^59^; Rejko Krueger, MD^2^; Mark Lew, MD^49^; Zoltan Mari, MD^56^; Connie Marras, PhD^45^; Maria Jose Martí, PhD^34^; Nikolaus McFarland, PhD^54^; Tiago Mestre, PhD^46^; Brit Mollenhauer, MD^8^; Emile Moukheiber, MD^28^; Alastair Noyce, PhD^63^ Wolfgang Oertel, PhD^64^; Njideka Okubadejo, MD^65^; Sarah O'Shea, MD^39^; Rajesh Pahwa, MD^48^; Nicola Pavese, PhD^57^; Werner Poewe, MD^7^; Ron Postuma, MD^55^; Giulietta Riboldi, MD^53^; Lauren Ruffrage, MS^18^; Javier Ruiz Martinez, PhD^35^; David Russell, PhD^1^; Marie H Saint‐Hilaire, MD^22^; Neil Santos, BS^51^; Wesley Schlett^47^; Ruth Schneider, MD^23^; Holly Shill, MD^50^; David Shprecher, DO^24^; Tanya Simuni, MD^3^; David Standaert, PhD^18^; Leonidas Stefanis, PhD^38^; Yen Tai, PhD^29^; Caroline Tanner, PhD^9^; Arjun Tarakad, MD^20^; Eduardo Tolosa PhD^34^ and Aleksandar Videnovic, MD^47^

**Coordinators**:

Susan Ainscough, BA^30^; Courtney Blair, MA^18^; Erica Botting^19^; Isabella Chung, BS^56^; Kelly Clark^24^; Ioana Croitoru^35^; Kelly DeLano, MS^32^; Iris Egner, PhD^7^; Fahrial Esha, BS^53^; May Eshel^36^; Frank Ferrari, BS^44^; Victoria Kate Foster^57^; Alicia Garrido, MD^34^; Madita Grümmer^59^; Bethzaida Herrera^50^; Ella Hilt^26^; Chloe Huntzinger, BA^52^; Raymond James, BS^22^; Farah Kausar, PhD^9^; Christos Koros, MD, PhD^38^; Yara Krasowski^60^; Dustin Le, BS^17^; Ying Liu, MD^52^; Taina M. Marques, PhD^2^; Helen Mejia Santana, MA^39^; Sherri Mosovsky, MPH^41^; Jennifer Mule, BS^25^; Philip Ng, BS^45^; Lauren O'Brien^48^; Abiola Ogunleye, PGDip^29^; Oluwadamilola Ojo, MD^65^; Obi Onyinanya, BS^28^; Lisbeth Pennente, BA^31^; Romina Perrotti^55^; Michael Pileggi, MS^55^; Ashwini Ramachandran, MSc^12^; Deborah Raymond, MS^14^; Jamil Razzaque, MS5^8^; Shawna Reddie, BA^46^; Kori Ribb, BSN,^28^; Kyle Rizer, BA^54^; Janelle Rodriguez, BS^27^; Stephanie Roman, HS^1^; Clarissa Sanchez, MPH^20^; Cristina Simonet, PhD^29^; Anisha Singh, BS^23^; Elisabeth Sittig^64^; Barbara Sommerfeld MSN^16^; Angela Stovall, BS^44^; Bobbie Stubbeman, BS^32^; Alejandra Valenzuela, BS^49^; Catherine Wandell, BS^21^; Diana Willeke^8^; Karen Williams, BA^3^ and Dilinuer Wubuli, MB^45^

Partners Scientific Advisory Board (Acknowledgement)

Funding: PPMI – a public‐private partnership – is funded by the Michael J. Fox Foundation for Parkinson's Research and funding partners, including 4D Pharma, Abbvie, AcureX, Allergan, Amathus Therapeutics, Aligning Science Across Parkinson's, AskBio, Avid Radiopharmaceuticals, BIAL, Biogen, Biohaven, BioLegend, BlueRock Therapeutics, Bristol‐Myers Squibb, Calico Labs, Celgene, Cerevel Therapeutics, Coave Therapeutics, DaCapo Brainscience, Denali, Edmond J. Safra Foundation, Eli Lilly, Gain Therapeutics, GE HealthCare, Genentech, GSK, Golub Capital, Handl Therapeutics, Insitro, Janssen Neuroscience, Lundbeck, Merck, Meso Scale Discovery, Mission Therapeutics, Neurocrine Biosciences, Pfizer, Piramal, Prevail Therapeutics, Roche, Sanofi, Servier, Sun Pharma Advanced Research Company, Takeda, Teva, UCB, Vanqua Bio, Verily, Voyager Therapeutics, the Weston Family Foundation and Yumanity Therapeutics.

^1^Institute for Neurodegenerative Disorders, New Haven, CT

^2^University of Luxembourg, Luxembourg

^3^Northwestern University, Chicago, IL

^4^University of Iowa, Iowa City, IA

^5^VectivBio AG

^6^The Michael J. Fox Foundation for Parkinson's Research, New York, NY

^7^Innsbruck Medical University, Innsbruck, Austria

^8^Paracelsus‐Elena Klinik, Kassel, Germany

^9^University of California, San Francisco, CA

^10^Laboratory of Neuroimaging (LONI), University of Southern California

^11^BioRep, Milan, Italy

^12^University of Pennsylvania, Philadelphia, PA

^13^National Institute on Aging, NIH, Bethesda, MD

^14^Mount Sinai Beth Israel, New York, NY

^15^Indiana University, Indianapolis, IN

^16^Emory University of Medicine, Atlanta, GA

^17^Oregon Health and Science University, Portland, OR

^18^University of Alabama at Birmingham, Birmingham, AL

^19^University of South Florida, Tampa, FL

^20^Baylor College of Medicine, Houston, TX

^21^University of Washington, Seattle, WA

^22^Boston University, Boston, MA

^23^University of Rochester, Rochester, NY

^24^Banner Research Institute, Sun City, AZ

^25^Cleveland Clinic, Cleveland, OH

^26^University of Tübingen, Tübingen, Germany

^27^University of California, San Diego, CA

^28^Johns Hopkins University, Baltimore, MD

^29^Imperial College of London, London, UK

^30^University of Salerno, Salerno, Italy

^31^Parkinson's Disease and Movement Disorders Center, Boca Raton, FL

^32^University of Cincinnati, Cincinnati, OH

^34^Hospital Clinic of Barcelona, Barcelona, Spain

^35^Hospital Universitario Donostia, San Sebastian, Spain

^36^Tel Aviv Sourasky Medical Center, Tel Aviv, Israel

^37^St. Olav's University Hospital, Trondheim, Norway

^38^National and Kapodistrian University of Athens, Athens, Greece

^39^Columbia University Irving Medical Center, New York, NY

^40^Stanford University, Stanford, CA

^41^University of Pittsburgh, Pittsburgh, PA

^42^Center for Strategy Philanthropy at Milken Institute, Washington D.C.

^43^12, New Brunswick, NJ

^44^University of Michigan, Ann Arbor, MI

^45^Toronto Western Hospital, Toronto, Canada

^46^The Ottawa Hospital, Ottawa, Canada

^47^Massachusetts General Hospital, Boston, MA

^48^University of Kansas Medical Center, Kansas City, KS

^49^University of Southern California, Los Angeles, CA

^50^Barrow Neurological Institute, Phoenix, AZ

^51^Mayo Clinic Arizona, Scottsdale, AZ

^52^University of Colorado, Aurora, CO

^53^NYU Langone Medical Center, New York, NY

^54^University of Florida, Gainesville, FL

^55^Montreal Neurological Institute and Hospital/McGill, Montreal, QC, Canada

^56^Cleveland Clinic‐Las Vegas Lou Ruvo Center for Brain Health, Las Vegas, NV

^57^Clinical Ageing Research Unit, Newcastle, UK

^58^John Radcliffe Hospital Oxford and Oxford University, Oxford, UK

^59^Universität Lübeck, Luebeck, Germany

^60^Radboud University, Nijmegen, Netherlands

^61^TransThera Consulting

^62^Duke University, Durham, NC

^63^Wolfson Institute of Population Health, Queen Mary University of London, UK

^64^Philipps‐University Marburg, Germany

^65^University of Lagos, Nigeria

## Supporting information


**Figure S1.** The set of 17 SPECT templates with a range of progressively reduced signal intensities in the Caudate and Putamen, corresponding to increased deficits observed as PD progresses, in MNI152 space (Montreal Neurological Institute). An optimal linear combination of these templates was determined for each subject as part of the spatial normalization process.
**Figure S2.** Results from the Level 2 regression analyses of the Head‐to‐Head [^18^F]AV133 PET and [^123^I]Ioflupane SPECT SBR data (top row) and transformation of the data into the Centamine scale (bottom row) for the five target regions of interest. This includes the addition of longitudinal scans to the baseline data presented in Figure 3. Note that a small number of points are outside the axis range of 0–130 in the Centamine plots and are not displayed.
**Figure S3.** Data from Data Sets 1 & 2 mapped into the Centamine scale following Level 1 & 2 analyses (Data Set 1 (HC), Data Set 2 (HTH), [^123^I]Ioflupane SPECT (Blue), [^18^F]AV133 PET (Green), group means displayed as red dots and black lines indicate +/− SD. HC = Healthy Controls, HTH = Head‐to‐Head). This includes the addition of longitudinal scans to the baseline data presented in Figure 4.
**Figure S4.** Bland–Altman plot of the Centamine values derived from the Level 2 regression analyses of the Head‐to‐Head [^18^F]AV133 PET and [^123^I]Ioflupane SPECT SBR data for the five target regions of interest.
**Figure S5.** Histogram of the Centamine values derived from the Level 2 regression analyses of the Head‐to‐Head [^18^F]AV133 PET and [^123^I]Ioflupane SPECT SBR data for the five target regions of interest.
**Figure S6.** Histogram of the differences in Centamine values between [^1^⁸F]AV133 PET and [^123^I]Ioflupane SPECT, based on Level 2 regression analyses of SBR data from the five target regions of interest

## Data Availability

Details on how individual research groups can obtain the necessary data used in this manuscript to calibrate their own analysis pipelines in Centamines can be found on the PPMI website (http://ppmi-info.org for details).
