# Supplementary material for: Toward Harmonizing Quantification of Dopamine Neuron Imaging Biomarkers in Parkinson's Disease: The Centamine Scale
Source: Ann Neurol. 2026 Jan 20;99(4):949–63. doi: 10.1002/ana.78116 (PMC13011789; doi:10.1002/ana.78116)
Supplement: Supplementary file 1 — Figure S1. The set of 17 SPECT templates with a range of progressively reduced signal intensities in the Caudate and Putamen, corresponding to increased deficits observed as PD progresses, in MNI152 space (Montreal Neurological Institute). An optimal linear combination of these templates was determined for each subject as part of the spatial normalization process. Figure S2. Results from the Level 2 regression analyses of the Head‐to‐Head [18F]AV133 PET and [123I]Ioflupane SPECT SBR data (top row) and transformation of the data into the Centamine scale (bottom row) for the five target regions of interest. This includes the addition of longitudinal scans to the baseline data presented in Figure 3. Note that a small number of points are outside the axis range of 0–130 in the Centamine plots and are not displayed. Figure S3. Data from Data Sets 1 & 2 mapped into the Centamine scale following Level 1 & 2 analyses (Data Set 1 (HC), Data Set 2 (HTH), [123I]Ioflupane SPECT (Blue), [18F]AV133 PET (Green), group means displayed as red dots and black lines indicate +/− SD. HC = Healthy Controls, HTH = Head‐to‐Head). This includes the addition of longitudinal scans to the baseline data presented in Figure 4. Figure S4. Bland–Altman plot of the Centamine values derived from the Level 2 regression analyses of the Head‐to‐Head [18F]AV133 PET and [123I]Ioflupane SPECT SBR data for the five target regions of interest. Figure S5. Histogram of the Centamine values derived from the Level 2 regression analyses of the Head‐to‐Head [18F]AV133 PET and [123I]Ioflupane SPECT SBR data for the five target regions of interest. Figure S6. Histogram of the differences in Centamine values between [1⁸F]AV133 PET and [123I]Ioflupane SPECT, based on Level 2 regression analyses of SBR data from the five target regions of interest [file ANA-99-949-s001.zip › 3_Online Supplementary File PPMI.pdf]

**Supplementary Appendix 1**

**Members of The Parkinson's Progression Markers Initiative (PPMI)**

Data used in the preparation of this article were obtained from the Parkinson's Progression Markers Initiative (PPMI) database (www.ppmi-info.org). The investigators within PPMI contributed to the design and implementation of PPMI and/or provided data and collected biospecimens but did not participate in the analysis or writing of this report.

The members of The Parkinson's Progression Markers Initiative (PPMI) are listed below.

**PPMI Executive Steering Committee**

Kenneth Marek, MD (Principal Investigator)
Tanya Simuni, MD
Andrew Siderowf, MD
Caroline Tanner, MD
Thomas F. Tropea, DO
Tatiana Foroud, PhD
Lana Chahine, MD
Brit Mollenhauer, MD
Kalpana Merchant, MD
Douglas Galasko, MD
Christopher Coffey, PhD
Kathleen Poston, MD
Roseanne Dobkin, PhD
Ethan Brown, MD
Roy Alcalay, MD
Dan Weintraub, MD
Emily Flagg, BA
Kimberly Fabrizio, BA

---

**PPMI Steering Committee**

Susan Bressman, MD
Cornelis Blauwendraat, PhD
Paola Casalin, PhD
Sonya Dumanis, PhD
Raymond James, RN
Karl Kieburtz, MD
Sneha Mantri, MS
Werner Poewe, MD

Michael Schwarzschild, MD
John Seibyl, MD
David Standaert, PhD
Duygu Tosun-Turgut, PhD

---

**Michael J. Fox Foundation**

Sohini Chowdhury, MA
Jamie Eberling, PhD
Mark Frasier, PhD
Leslie Kirsch, EdD
Katie Kopil, PhD
Maggie Kuhl, BA
Alyssa O'Grady, BA
Todd Sherer, PhD
Tawny Willson, MBS

---

**PPMI Study Cores**

**Project Management Core**
Emily Flagg, BA

**Site Management Core**
Tanya Simuni, MD
Bridget McMahon, BS

**Data Strategy and Technical Operations**
Craig Stanley, PhD
Kimberly Fabrizio, BA

**Data Management Core**
Dixie Ecklund, MBA, MSN
Christine Kohnen, PhD

**Screening Core**
Tatiana Foroud, PhD
Laura Heathers, BA
Christopher Hobbick, BSCE
Gena Antonopoulos, BSN

**Imaging Core**
John Seibyl, MD
Kathleen Poston, MD

**Statistics Core**
Christopher Coffey, PhD
Chelsea Caspell-Garcia, MS
Michael Brumm, MS

**Bioinformatics Core**
Arthur Toga, PhD
Karen Crawford, MLIS

**Biorepository Core**
Tatiana Foroud, PhD
Jan Hamer, BS

**Biologics Review Committee**
Brit Mollenhauer, MD
Douglas Galasko, MD
Kalpana Merchant, MD

**Genetics Core**
Andrew Singleton, PhD

**Pathology Core**
Tatiana Foroud, PhD
Dirk Keene, MD

**FOUND**
Caroline Tanner, MD
Ethan Brown, MD

**PPMI Online**
Carlie Tanner, MD
Ethan Brown, MD
Lana Chahine, MD
Roseanne Dobkin, PhD
Monica Korell, MPH

---

**PPMI Site Investigators**

Neha Prakash, MD
Tanya Simuni, MD
Nabila Dahodwala, MD
Caroline Tanner, MD
Lana Chahine, MD
Brit Mollenhauer, MD

Sebastian Schade, MD
Douglas Galasko, MD
Anat Mirelman, PhD
Roy Alcalay, MD
Katherine Leaver, MD
Marie Saint-Hilaire, MD
Ruth Schneider, MD
Christopher Tarolli, MD
Werner Poewe, MD
Aleksandar Videnovic, MD
David Standaert, PhD
Marissa Dean, MD
Sonja Jonsdottir, PhD
Rejko Krueger, MD
Claire Pauly, PhD
Stewart Factor, DO
Penelope Hogarth, MD
Robert Hauser, MD
Amy Amara, PhD
Michelle Fullard, MD
Cyrus Zabetian, MD
Hubert Fernandez, MD
Kathrin Brockmann, MD
Isabel Wurster, PhD
Yen Tai, PhD
Paolo Barone, PhD
Marina Picillo, MD
Stuart Isaacson, MD
Alberto Espay, MD
Eduardo Tolosa, PhD
Javier Ruiz Martinez, PhD
Leonidas Stefanis, PhD
Kelvin Chou, MD
Lorraine Kalia, MD
Connie Marras, PhD
David Grimes, MD
Tiago Mestre, PhD
Rajesh Pahwa, MD
Mark Lew, MD
Holly Shill, MD
Shyamal Mehta, MD

Giulietta Riboldi, MD
Nikolaus McFarland, PhD
Ron Postuma, MD
Zoltan Mari, MD
David Ledingham, MD
Nicola Pavese, PhD
Michele Hu, PhD
Norbert Brueggemann, MD
Christine Klein, MD
Bastiaan Bloem, PhD
Cristina Simonet, PhD
Alastair Noyce, PhD
Anette Janzen, PhD
David Pedrosa, MD
Wolfgang Oertel, PhD
Njideka Okubadejo, MD
David Shprecher, DO
Arjun Tarakad, MD
Emile Moukheiber, MD

---

**PPMI Site Coordinators**

Joy Antala
Carla Aranda
Karen Williams
Sophia Melton
Karina Benson
Ashwini Ramachandran
Danielle Potts
Grace LaMoure
Ritikha Vengadesh
Ryan Manzler
Jaime Heller
Primi Ranola
Farah Kausar
Sherri Mosovsky
Diana Willeke
Elizabeth Kalinkara-Gomez
Janelle Rodriguez
Nobuko Kemmotsu

May Eshel
Deborah Raymond
Abigail Desrosiers
Raymond James
Lauren Jackson
Iris Egner
Wesley Schlett
Courtney Blair
Lauren Ruffrage
Berenice Sevilla
Barbara Sommerfeld
Dustin Le
Erica Botting
Gabriella Mazur
Daniele Derlein
Evan Doll
Ying Liu
Ciera Cobb
Olivia Masiewicz
Jennifer Mule
Michael Morsillo
Ella Hilt
Aldazier Jakiran
Dominga Valentino
Lisbeth Pennente
Bobbie Stubbeman
Alicia Garrido
Valeria Ravasi
Ioana Croitoru
Christos Koros
Nikolas Papagiannakis
Frank Ferrari
Mengyu Zheng
Shawna Reddie
Alicia Alejandra
Andrea Gray
Alejandra Valenzuela
Caitlin Goodman
Sara Dresler
Neil Santos
Fahrial Esha

Kyle Rizer
Nadine Zablith
Liliana Dumitrescu
Debra Galley
Victoria Kate Foster
Jamil Razzaque
Madita Grümmer
Yara Krasowski
Natalie Donkor
Elisabeth Sittig
Oluwadamilola Ojo
Kelly Clark
Rory Mahabir
Kori Ribb
Shamera Willoughby
