# Supplementary material for: Toward Harmonizing Quantification of Dopamine Neuron Imaging Biomarkers in Parkinson's Disease: The Centamine Scale
Source: Ann Neurol. 2026 Jan 20;99(4):949–63. doi: 10.1002/ana.78116 (PMC13011789; doi:10.1002/ana.78116)

Supplementary Figure 1

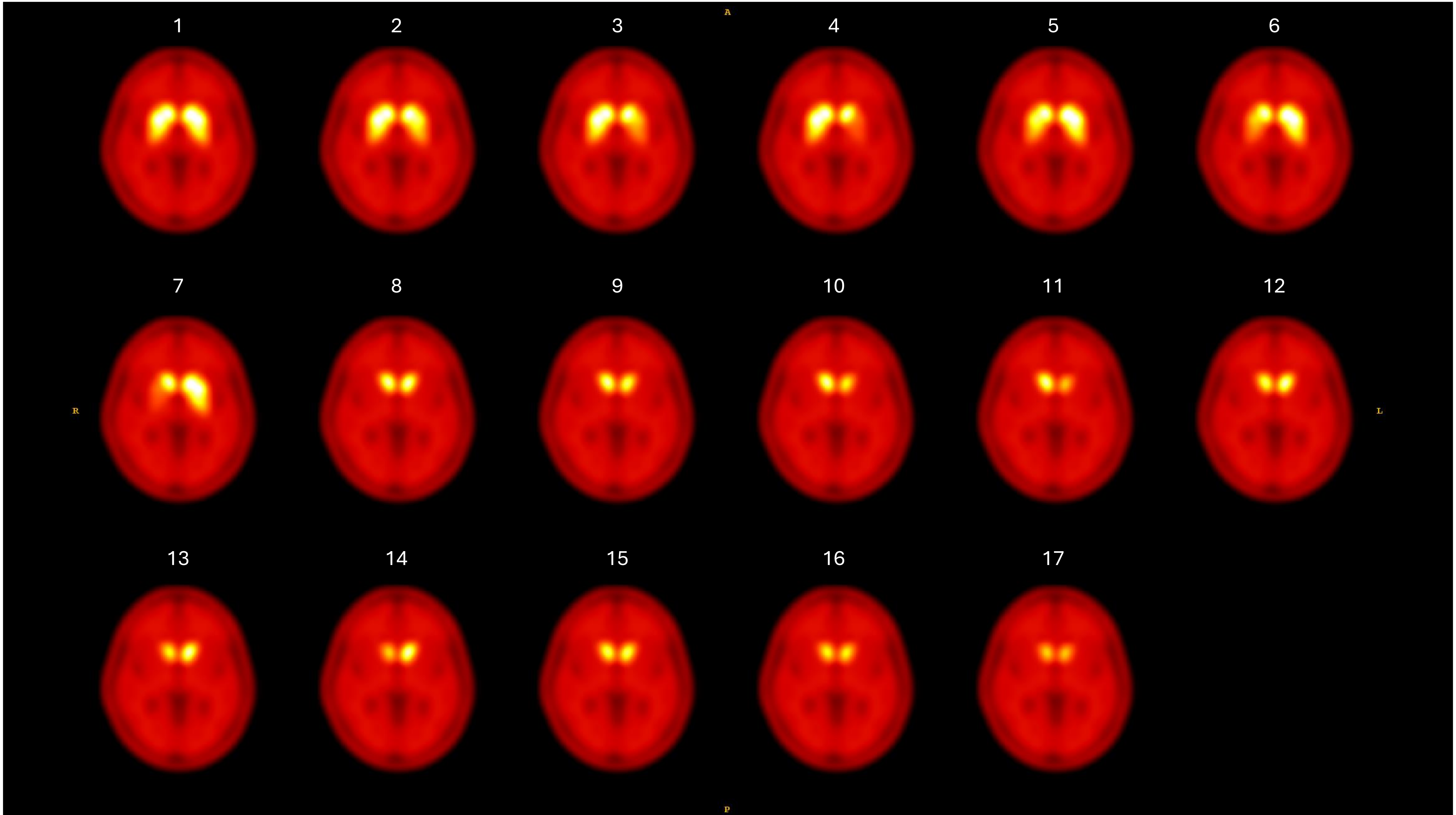

Supplementary Figure 2

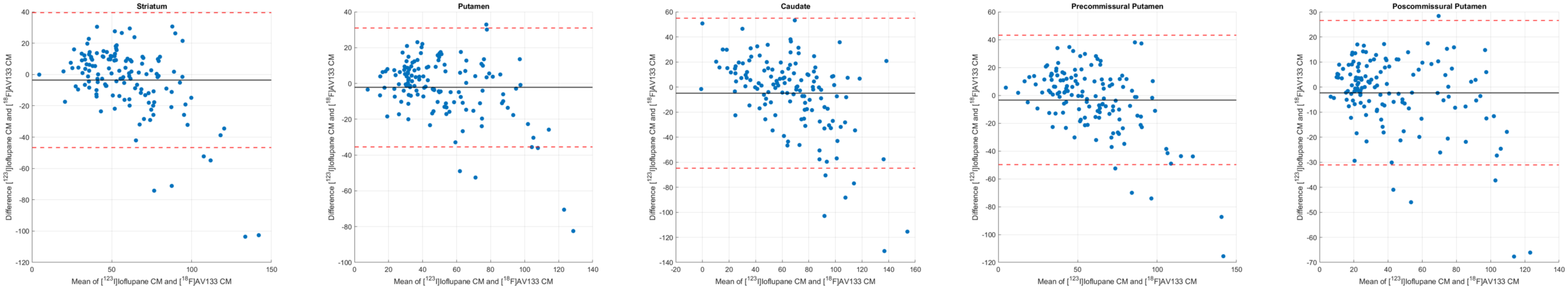

Supplementary Figure 3

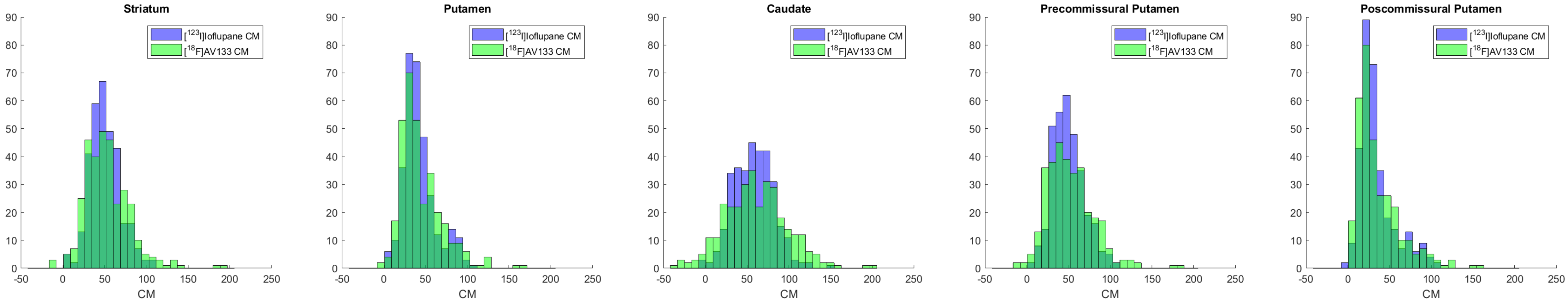

Supplementary Figure 4

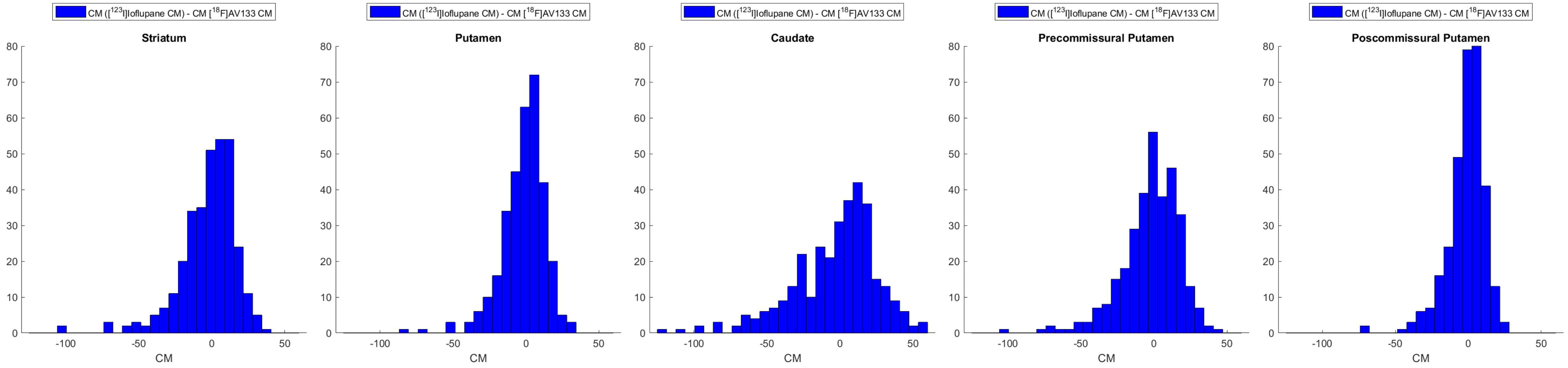

Supplementary Figure 5

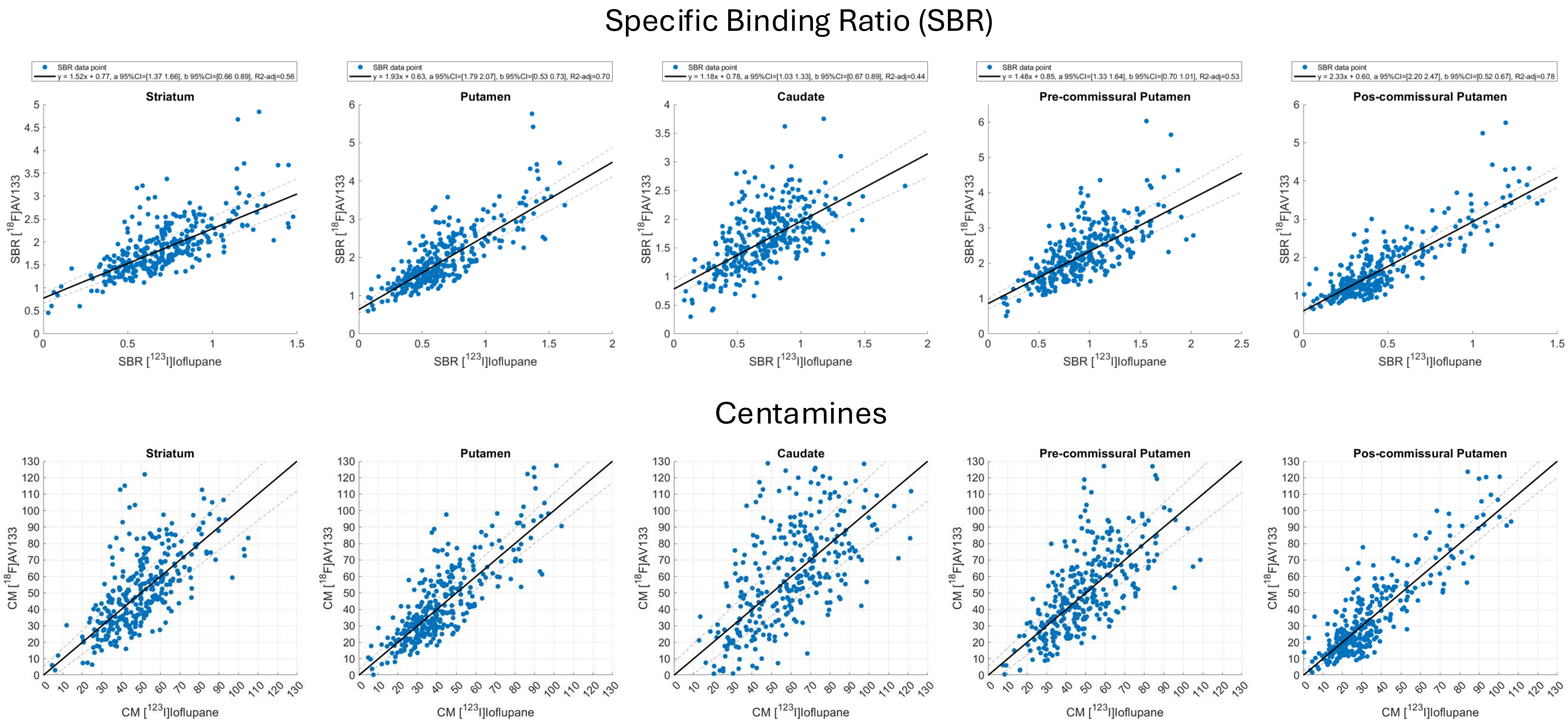

Supplementary Figure 6

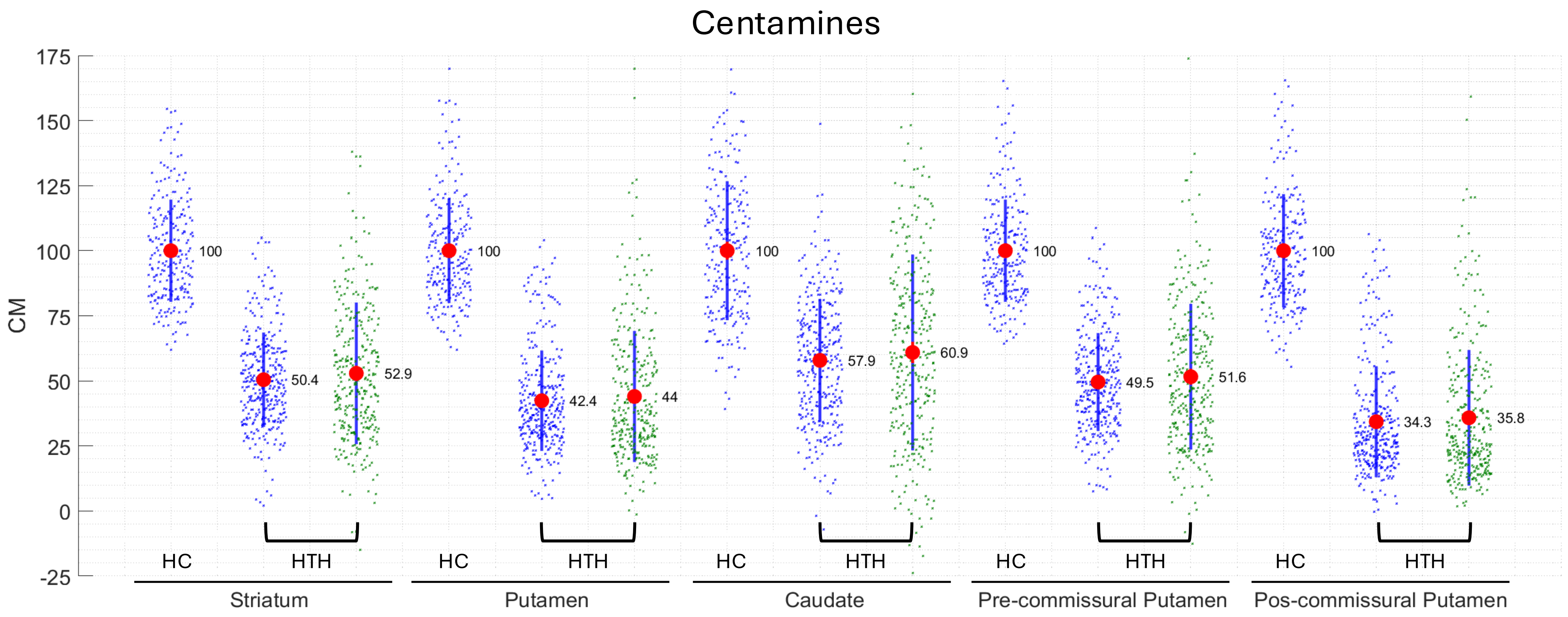

Supplement: Supplementary file 1 — Figure S1. The set of 17 SPECT templates with a range of progressively reduced signal intensities in the Caudate and Putamen, corresponding to increased deficits observed as PD progresses, in MNI152 space (Montreal Neurological Institute). An optimal linear combination of these templates was determined for each subject as part of the spatial normalization process. Figure S2. Results from the Level 2 regression analyses of the Head‐to‐Head [18F]AV133 PET and [123I]Ioflupane SPECT SBR data (top row) and transformation of the data into the Centamine scale (bottom row) for the five target regions of interest. This includes the addition of longitudinal scans to the baseline data presented in Figure 3. Note that a small number of points are outside the axis range of 0–130 in the Centamine plots and are not displayed. Figure S3. Data from Data Sets 1 & 2 mapped into the Centamine scale following Level 1 & 2 analyses (Data Set 1 (HC), Data Set 2 (HTH), [123I]Ioflupane SPECT (Blue), [18F]AV133 PET (Green), group means displayed as red dots and black lines indicate +/− SD. HC = Healthy Controls, HTH = Head‐to‐Head). This includes the addition of longitudinal scans to the baseline data presented in Figure 4. Figure S4. Bland–Altman plot of the Centamine values derived from the Level 2 regression analyses of the Head‐to‐Head [18F]AV133 PET and [123I]Ioflupane SPECT SBR data for the five target regions of interest. Figure S5. Histogram of the Centamine values derived from the Level 2 regression analyses of the Head‐to‐Head [18F]AV133 PET and [123I]Ioflupane SPECT SBR data for the five target regions of interest. Figure S6. Histogram of the differences in Centamine values between [1⁸F]AV133 PET and [123I]Ioflupane SPECT, based on Level 2 regression analyses of SBR data from the five target regions of interest [file ANA-99-949-s001.zip › 3_Supplementary_Figures.pdf]
